# Diversity of culturable bacteria including *Pantoea* in wild mosquito *Aedes albopictus*

**DOI:** 10.1186/1471-2180-13-70

**Published:** 2013-03-27

**Authors:** Claire Valiente Moro, Florence Hélène Tran, Fara Nantenaina Raharimalala, Pierre Ravelonandro, Patrick Mavingui

**Affiliations:** 1 Université de Lyon, Lyon, F-69622, France; 2 Ecologie Microbienne, CNRS UMR 5557, INRA USC1364, VetAgroSup,, Villeurbanne, France; 3 Département d’Entomologie de la Faculté des Sciences d’Antananarivo, Antananarivo, Madagascar; 4 Centre National de Recherche sur l′Environnement, Antananarivo, Madagascar; 5 Present address: Medical Entomology Unit, Institut Pasteur de Madagascar, Antananarivo, Madagascar

**Keywords:** Culturable bacteria, Asian tiger mosquito, *Pantoea*, Plasmids

## Abstract

**Background:**

The microbiota has been shown to play an important role in the biology of insects. In recent decades, significant efforts have been made to better understand the diversity of symbiotic bacteria associated with mosquitoes and assess their influence on pathogen transmission. Here, we report the bacterial composition found in field-caught *Aedes albopictus* populations by using culture-dependent methods.

**Results:**

A total of 104 mosquito imagos (56 males and 48 females) were caught from four contrasting biotopes of Madagascar and their bacterial contents were screened by plating whole body homogenates on three different culture media. From 281 bacterial colony types obtained, amplified ribosomal DNA restriction analysis (ARDRA) showed they had 40 distinct ribotypes. Sequencing and BLAST analysis of the 16S rDNA genes responsible for each representative profile made it possible to identify 27 genera distributed in three major phyla. In female mosquitoes, bacterial isolates were mostly Proteobacteria (51.3%) followed by Firmicutes (30.3%) and Actinobacteria (18.4%). Conversely, Actinobacteria was the most abundant phylum in male mosquitoes (48%) followed by Proteobacteria (30.6%) and Firmicutes (20.4%). The relative abundance and composition of isolates also varied between sampling sites, ranging from 3 distinct families in Ankazobe to 8 in Tsimbazaza Park, and Toamasina and Ambohidratrimo. *Pantoea* was the most common genus in both females and males from all sampling sites, except for Ambohidratrimo. No differences in genome size were found between *Pantoea* isolates from mosquitoes and reference strains in pulse field gel electrophoresis. However, according to the numbers and sizes of plasmids, mosquito isolates clustered into three different groups with other strains isolated from insects but distinct from isolates from the environment.

**Conclusions:**

The recent upsurge in research into the functional role of the insect microbiota prompts the interest to better explore the role some bacteria detected here may have in the mosquito biology. Future studies of culturable bacteria might decipher whether they have a biological role in the invasiveness of *Ae. albopictus*. As a possible candidate for paratransgenesis, the predominant genus *Pantoea* will be characterized to better understand its genetic contents and any possible influence it may have on vector competence of *Ae. albopictus*.

## Background

Insects can be considered as holobiont units in which the insect host and its microbiota are involved in complex reciprocal multipartite interactions [1]. Numerous studies have shown the beneficial impact of microbiota on their insect hosts, especially in phytophagous insects. For instance, bacterial endosymbionts contribute to different biological functions like supplying essential nutrients, inducing resistance to pathogens and parasitoids, and conferring tolerance of temperature stress [2-6]. Surprisingly, the nature and function of naturally occurring microorganisms harboured by hematophagous arthropods have been largely overlooked in research even though these aspects might be relevant in the study of pathogen transmission. There are nevertheless a few examples of the molecular characterization of bacterial species in the microbiota of mosquito vectors based on culture-dependent or independent methods or both [7-12]. Recent years have seen a growing interest in metagenomic-based studies of bacterial communities possibly displacing traditional culture-based analysis [13]. For instance, next generation sequencing technology was successfully used in *Anopheles gambiae* to provide a ‘deeper’ description of the bacterial community than can be achieved with conventional molecular techniques [14]. However, even though such an approach can reveal the number and richness of bacterial species, it is still important to search for culturable bacteria residing in insects for several reasons. Culturing bacteria still offers the best way of observing the diverse characteristics of the isolated organism. The physiological characteristics of bacterial isolates need to be determined to investigate properties such as antibiotic resistance, interspecies growth inhibition or population dynamics within mosquito cohorts. The availability of key representative isolates therefore allows detailed analyses of biochemical, metabolic and functional processes. For example, isolation of Actinobacteria showed that they are involved in cellulose and hemicellulose degradation pathways in termites [15,16]. Culturable Proteobacteria associated with insects were shown to play a role in carbohydrate degradation and nutrient provision [17,18]. In addition to phenotypic characterization of bacterial isolates, culturing also facilitates bacterial genome sequencing, a further link towards revealing functionality [19]. There have also been a number of recent studies of the use of engineered bacteria in the development of more efficient insect control strategies. Insect bacterial symbionts were genetically modified and the recombinants reintroduced into their native host. This paratransgenesis strategy involves different steps but requires the initial isolation of a suitable bacterial species [20]. In mosquitoes, paratransgenesis studies have mainly focused on anopheline mosquitoes, vectors of the malaria parasite [11]. As an efficient colonizer of *Anopheles stephensi*, the bacterium *Asaia* sp. was originally proposed as a candidate for malaria control [21], but recently it has been suggested that *Pantoea agglomerans*, another bacterial symbiont of *Anopheles*, could also be engineered to express and secrete anti-*Plasmodium* effector proteins [22].

Screening culturable bacteria using traditional microbiological techniques is an important method in mosquito-associated microbiota investigation. One of the key mosquito species for pathogen transmission is *Aedes albopictus*, which is a vector of several arboviruses pathogenic to humans, some having a devastating impact worldwide [23]. This species has been identified as the primary vector responsible for recent outbreaks of Dengue and Chikungunya which emerged in Madagascar and other neighbouring islands [24,25]. Until now, no bacterial species has been reported as being essential for mosquito biology, while only *Wolbachia* has been proposed as a gene driver system in *Aedes* mosquitoes. Here we present an in-depth investigation of culturable bacteria in natural populations of *Ae. albopictus*. Our main objective was to assess the abundance and phylogenetic diversity of culturable bacteria in a set of adult male and female mosquitoes from different regions of Madagascar. This deeper screening of the bacterial isolates retrieved significantly extends our previous work on the prevalence of *Acinetobacter* and *Asaia* associated with Madagascarian populations of *Ae. albopictus*[26].

## Methods

### Sampling areas and mosquito collection

The sampling areas and capture procedure were approved by Madagascar National Parks. *Aedes albopictus* specimens were sampled in December 2010 at four sites in two regions of Madagascar, Analamanga and Antsinanana. The main characteristics of the sampling sites are summarized in Table 1. Briefly, the two regions have a similar tropical climate, but different biotopes according to the vegetation or the presence of human or animal hosts susceptible to mosquito bites. Butterfly netting was used to collect both female and male mosquitoes flying near the grass or ground, as previously described [27]. The live mosquitoes collected were identified using morphological characteristics keys [28] and transported to the local laboratory.

**Table 1:** **Ecological characteristics of *Ae. albopictus *sampling sites**

**Region**	**Site**	**Zone**	**Vegetation**	**Potential hosts**	*Male	*Female
Analamanga	Ambohidratrimo	Village outskirts	Bamboo hedge	Humans, birds, reptiles	20	5
	Tsimbazaza Park	City	Bushes and fruit trees (mango)	Humans, lemurs, reptiles, birds	7	8
	Ankazobe	Village outskirts	Bamboo forest	Humans, chickens	13	19
Atsinanana	Toamasina Town	City	Bushes and fruit trees (banana tree)	Humans, chickens, ducks	16	16

*Numbers of mosquito individuals collected at each site in December 2010.

### Enrichment and isolation of bacteria from mosquitoes

Only non-blooded mosquitoes were used for the analysis. Specimens of *Ae. albopictus* were anaesthetised with ether and surface-disinfected as previously described [12], then crushed individually in 150 μl of sterile 0.8% NaCl with sterile piston pellets. After a brief vortexing, the homogenate was used in different isolation procedures using various media, from generalist to selective. All solid media were supplemented with 2.5 μg ml^-1^ amphotericin B to prevent the growth of fungi. An aliquot of the homogenate (10 μl) was streaked onto a modified rich solid Luria-Bertani medium (LBm, LB with 5 mg ml^-1^ NaCl) and incubated at 28°C for 24 to 48 h. Another aliquot (20 μl) was inoculated into 1 ml of selective enrichment medium I (0.2% KNO_3_, 0.02% MgSO_4_.7H_2_O, 0.2% sodium acetate, 0.04 M KH_2_PO_4_, pH 6), a medium which is suitable for the isolation of *Acinetobacter* species [29]. Cultures were incubated at 30°C for 24 to 48 h with shaking. When microbial growth occurred, an aliquot (10 μl) of the culture was streaked onto Herellea agar plates (Biolife, Italy), a medium suitable for the isolation of Gram-negative bacteria especially members of the *Acinetobacter* genus and the *Enterobacteriaceae* family [30]. These cultures were further incubated at 37°C for 24 to 48 h. In parallel, 1 ml of pre-enrichment liquid medium (pH 3.5), which is suitable for the isolation of acetic acid bacteria [31], was inoculated with an aliquot of homogenate (20 μl). These cultures were incubated with shaking at 30°C for 3 days. When microbial growth occurred, an aliquot (10 μl) was streaked onto CaCO_3_ agar plates (pH 6.8), a medium suitable for the isolation of members of the genus *Asaia*, and the plate was incubated at 30°C for 3 days as previously described [32]. Colonies were selected according to various characteristics including colour, shape, or size. Individual colonies were then re-inoculated onto fresh agar plates of the appropriate isolation medium. Newly formed colonies were streaked again to check for purity and stored in 25% glycerol at -20°C for two weeks before they were transported to the laboratory in Lyon, France. Isolates were re-streaked and new glycerol stocks were made and stored at -80°C. Brief morphological descriptions of colony size, shape and colour were recorded for each isolate.

### PCR and amplified ribosomal DNA restriction analysis (ARDRA)

For PCR, a sterile toothpick was used to transfer bacteria from a single colony freshly grown on appropriate medium into 20 μl sterile water in a 0.5 ml Eppendorf tube. The homogenate was placed on a heating block at 95°C for 2 min followed by 2 min on ice. This step was repeated and the tube was centrifuged at 16,000 g for 5 min. The supernatant (2 μl) was used as template in a 50-μl PCR reaction. Bacterial 16S *rrs* genes were amplified with eubacterial-specific primers pA (5^′^-AGAGTTTGATCCTGGCTCAG-3^′^) and pH (5^′^-AAGGAGGTGATCCAGCCGCA-3^′^) in reaction mixture containing 1 × polymerase reaction buffer, 0.2 μM MgCl_2_, 200 μM of each deoxynucleoside triphosphate, 10 pmol of each primer and 1 U of Taq polymerase (Invitrogen). PCR amplifications consisted of 3 min at 95°C, 35 cycles of 30 sec at 94°C, 40 sec at 55°C and 1 min 30 sec at 72°C, and finally 10 min at 72°C. Amplified DNA fragments were purified using the QIAquick PCR Purification Kit (Qiagen). ARDRA was performed to screen the *rrs* genes of bacterial isolates in 20 μl reactions containing 200 ng of DNA template, 1 × Buffer Tango™ and 10 U each of endonucleases RsaI and HhaI (Fermentas, France), as previously described [12]. DNA fragments were separated on 2% agarose gels stained with ethidium bromide with a 50-bp DNA ladder marker (Fermentas). Isolates showing the same restriction pattern with the two endonucleases were considered to be similar.

### Sequencing of *rrs* rRNA genes and phylogenetic analyses

Both strands of 16S rDNA amplified from isolates representative of each ARDRA profile were sequenced at Biofidal-DTAMB (FR Bio-Environment and Health, Lyon, France). Sequences were manually curated and assembled from forward and reverse primer-generated sequences. Curated sequences were then compared to available bacterial sequences in GenBank using the BLASTn program in the National Center for Biotechnology Information (http://blast.ncbi.nlm.nih.gov/Blast.cgi). The Ribosomal Database Project II Chimera Check was used (http://wdcm.nig.ac.jp/RDP/html/analyses.html) to discard any chimeric sequences.

Phylogenetic analyses were performed on a set of *Pantoea* sequences. Sequences of 16S rRNA genes from *Pantoea* isolates from mosquitoes were compared to all available sequences of *Pantoea* retrieved from GenBank that originated from other insect species and environments. Sequences were aligned using ClustalW then corrected manually using Bioedit software [33]. The resulting alignment was used to construct a maximum-likelihood tree using Seaview v.4.2.12. (http://pbil.univ-lyon1.fr/software/seaview.html). The tree topology was tested by bootstrap analysis with 1,000 resamplings.

### Pulse field gel electrophoresis (PFGE) of bacterial genomes

Undigested genomes of *Pantoea* isolates were analysed by PFGE according to published protocols with some modifications [26,34]. Briefly, isolates were grown in 10 ml of LBm liquid medium for 18 h at 30°C. Cell cultures were centrifuged at 5,000 g for 20 min at 4°C. The pellet was resuspended in 1 ml of 1 × Tris-EDTA buffer to obtain an optical density between 1.8 and 2.0. Cell suspensions (0.5 ml) were mixed volume to volume with 1.6% low melting point agarose (Biorad) and the mixture was distributed per 0.1 ml in the plug molds (Biorad) and cooled at 4°C. Cells were lysed in lysis solution (2 × Tris NaCl EDTA, 10% sodium lauroyl sarcosinate, 1.4 mg ml^-1^ lysozyme) at 37°C for 24 h and proteins were digested with proteinase K (Euromedex) in 0.5 M EDTA pH.8 containing 1% N-lauryl-sarcosine at 37°C for 48 h. The agarose plugs were incubated in 0.4 mg ml^-1^ phenylmethylsulfonyl fluoride (Sigma-Aldrich) at 50°C for 1 h, washed in 0.5 M EDTA pH.8 and electrophoresed in 0.8% chromosomal-grade agarose in 1 × TAE buffer using a CHEF Mapper XA (Biorad, France) at 14°C, a constant pulse of 500 ms and a field angle of 106° for 48 h at 3 V cm^-1^.

### Plasmid content

The procedure of Eckhardt [35] was used to identify high molecular weight plasmids in *Pantoea* as already described [36]. Briefly, 300 μl of bacterial culture (OD_600 nm_ equal to 0.5) was placed on 0.3% sodium lauroyl sarcosinate in 1 × Tris-borate-EDTA (TBE) buffer. After centrifugation at 2,300 *g* for 5 min at 4°C, the pellet was resuspended in 25 μl of lysis solution (9% saccharose, 1.9 mg ml^-1^ Lysozyme and 0.38 mg ml^-1^ RNase) and homogenates were loaded into 0.75% agarose gels in TBE containing 1% SDS. Electrophoresis was carried out at 10 V for 20 min then 85 V for 210 min. To identify lower-molecular-weight plasmids, a second method was used as described previously [37]. Plasmid sizes were estimated by comparing their relative mobility in agarose gels with those of plasmids from sequenced *Azospirillum* genomes [38,39], standard supercoiled plasmids (Life Technologies, Inc., USA) and two reference strains of *Pantoea* (*Pantoea stewartii* CFBP 3614 and *Pantoea agglomerans* CFBP 4740) retrieved from the French collection of phytopathogenic bacteria (http://www-intranet.angers.inra.fr/cfbp/).

### Statistical analysis

Differences between mosquito genders were tested by a chi-square test using R software [40].

## Results

### Bacterial diversity in *Ae. albopictus* from Madagascar

Culturable bacteria from 104 field-caught *Ae. albopictus* adults (56 males and 48 females) were analysed by plating homogenates of whole mosquito bodies onto different culture media. The bacterial isolates obtained from each mosquito were first screened on the basis of colony characteristics including colony size, shape, colour, margin, opacity, and elevation consistency. Only one colony per type was selected per plate, with the result that 62 colonies were selected from Herellea medium, 70 from CaCO_3_ medium and 149 from LBm giving a total of 281 colonies to analyse from the initial 3,000 isolates.

The 16S rRNA genes were amplified from these 281 isolates and analysed by ARDRA. Forty distinct ARDRA profiles were obtained. For each profile the 16S rRNA gene was sequenced from one or more randomly chosen isolates (Table 2). The sequences were analysed by BLASTn showing that they originated from 27 bacterial genera. Some genera exhibited identical ARDRA profiles with the two enzymes used. All the genera belonged to three major phyla: Actinobacteria, Firmicutes and Proteobacteria (see Table 2 for details of families, genera and species in each phylum). One isolate was affiliated with the Deinococcus-Thermus phylum.

**Table 2:** **Taxonomic affiliation and abundance of bacterial isolates from *Aedes albopictus***^***a***^

**Phylum**	**Class**	**Family**	**Nearest genus/species according to BLASTn**^**b**^	**% identity**	**GenBank accession**	**Isolates**^**c**^	
						**Male**	**Female**
Actinobacteria		*Dermacoccaceae*	*Dermacoccus* sp.	97	JQ958854	2	1
		*Intrasporangiaceae*	*Arsenicicoccus bolidensis*	97	JQ958843	1	0
			*Terrabacter* sp.	99	JQ958845	3	0
		*Microbacteriaceae*	*Curtobacterium flaccumfaciens*	98	JQ958832	5	1
			*Leucobacter* sp.	98	JQ958851	1	0
			*Microbacterium arborescens*	98	JQ958831	1	2
			*Microbacterium esteraromaticum*	99	JQ958857	0	1
			*Microbacterium flavescens*	98	JQ958839	0	1
		*Micrococcaceae*	*Arthrobacter albidus*	98	JQ958866	2	1
			*Kocuria* sp.	96	JQ958850	18	5
			*Micrococcus pumilus*	99	JQ958852	6	0
			*Micrococcus* sp.	98	JQ958858	6	1
		*Promicromonosporaceae*	*Cellulosimicrobium cellulans*	99	JQ958841	1	0
		*Streptomycetaceae*	*Streptomyces* sp.	99	JQ958882	1	1
Deinococcus Thermus		*Deinococcaceae*	*Deinococcus* sp.	99	JQ958848	1	0
Firmicutes		*Bacillaceae*	*Bacillus isronensis*	98	JQ958844	0	1
			*Bacillus megaterium*	99	JQ958856	0	1
			*Bacillus pumilus*	99	JQ958852	4	3
			*Bacillus* sp.	99	JQ958862	5	6
			*Bacillus* sp. KZ_AalM_Mm2	98	JQ958871	0	1
			*Bacillus subtilis*	97	JQ958867	0	1
		*Planococcaceae*	*Planococcus* sp.	99	JQ958846	1	0
		*Staphylococcaceae*	*Staphylococcus epidermidis*	98	JQ958849	0	1
			*Staphylococcus warneri*	99	JQ958869	10	9
Proteobacteria	α-Proteobacteria	*Rhodobacteraceae*	*Haematobacter massiliensis*	96	JQ958833	2	2
		*Rhodospirillaceae*	*Skermanella aerolata*	99	JQ958840	1	0
		*Sphingomonadaceae*	*Sphingomonas yunnanensis*	99	JQ958865	0	1
	β-Proteobacteria	*Neisseriaceae*	Uncultured *Neisseria* sp.	95	JQ958870	1	0
	γ-Proteobacteria	*Acetobacteraceae*	*Asaia* sp.	100	JQ958879	0	1
		*Enterobacteriaceae*	*Citrobacter freundii*	95	JQ958872	0	1
			*Enterobacter* sp.	99	JQ958885	1	3
			*Klebsiella oxytoca*	99	JQ958855	1	2
			*Pantoea* sp.	96	JQ958828	19	26
			*Acinetobacter baumannii*	100	JQ408698	0	3
		*Moraxellaceae*	*Acinetobacter lwoffii*	99	JQ408696	2	0
			*Pseudomonas oryzihabitans*	99	JQ958874	1	0
		*Pseudomonadaceae*	*Pseudomonas* sp.	99	JQ958861	1	0
		*Xanthomonadaceae*	*Xanthomonas* sp.	99	JQ958860	1	0

^a^ Sequence analyses are based on 1.3 to 1.5 kb of 16S rRNA genes and were performed in February 2013.

^b^ Best BLAST hit with a sequence having a species or genus name.

^c^ Number of isolates from each mosquito gender.

The distribution of bacterial phyla was significantly different according to mosquito gender (*P* = 0.0002). Most bacterial isolates from females were Proteobacteria (51.3%) followed by Firmicutes (30.3%) then Actinobacteria (18.4%). Conversely, Actinobacteria was the most abundant phylum in male mosquitoes (48%) followed by Proteobacteria (30.6%) and Firmicutes (20.4%). Some bacterial genera were found in both females and males, namely *Curtobacterium flaccumfaciens*, *Microbacterium*, *Arthrobacter*, *Kocuria*, *Streptomyces*, *Bacillus*, *Staphylococcus*, *Haematobacter massiliensis*, *Enterobacter*, *Klebsiella oxytoca*, *Acinetobacter* and *Pantoea*. Some bacterial genera were only associated with one mosquito gender. For instance, *Asaia*, *Citrobacter freundii* and *Sphingomonas* were only present in females whereas *Arseniococcus bolidensis*, *Cellulosimicrobium, Deinococcus, Leucobacter, Planococcus, Pseudomonas, Skermanella aerolata*, *Xanthomonas* and the *Neisseria* (referred to as being unculturable in databases) were exclusively isolated from males. According to the number of affiliated sequences, *Pantoea* was the most abundant genera, representing 25.8% of the total isolates from both male and female mosquitoes (Table 2).

### Relative abundance of bacterial isolates differs according to geographic distribution

**Figure 1:**
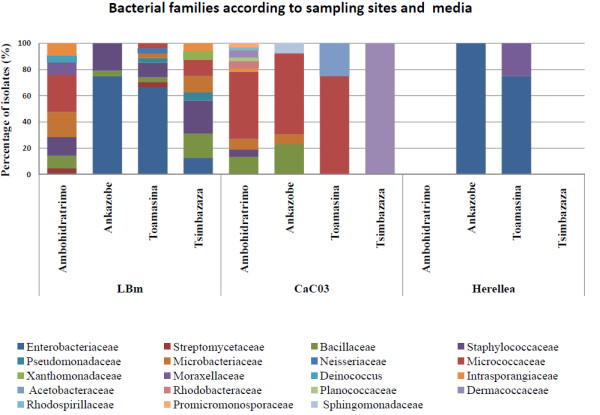
**Frequency of culturable isolates from field populations of *Ae. albopictus *according to sampling site and isolation medium.**

The relative abundance of isolates according to the sampling sites and the isolation media is shown in Figure 1. As expected, the isolation procedure using rich LBm medium gave the most diverse bacterial composition ranging from 3 to 8 distinct families per sampling site. Mosquitoes sampled in Ankazobe harboured only 3 bacterial families (*Enterobacteriaceae*, *Bacillaceae*, and *Staphylocacceae*), whereas mosquitoes from the other three sites (Tsimbazaza Park, Toamasina and Ambohidratrimo) harboured a total of 8 bacterial families per site. However, the abundance and composition of the bacteria from particular families varied between sampling sites. For instance, members of the families *Moraxellaceae* and *Deinococcaceae* were only isolated from mosquitoes in Ambohidratrimo, and those of the families *Neisseriaceae* and *Xanthomonadaceae* only from mosquitoes in Toamasina and Tsimbazaza park, respectively. While the isolation procedure was initially used to enrich for *Asaia*, isolates on CaCO_3_ medium largely belonged to Actinobacteria, irrespective of the origin of mosquitoes. Differences were also observed for members of the family *Acetobacteraceae* found in mosquitoes from Toamasina. As expected, on Herellea medium Gammaproteobacteria were detected with a majority of *Enterobacteriaceae* as well as bacteria of the genus *Acinetobacter.* These bacteria were only noted in mosquitoes from Toamasina and Ankazobe. Overall, the Ambohidratrimo mosquitoes harboured the highest number of distinct bacterial taxa with a total of 10 families in comparison to mosquitoes from other sites, which exhibited no more than 4 families. Members of the families *Staphylococcaceae*, *Rhodobacteraceae*, *Planoccoccaeae*, *Intrasporangiaceae*, *Rhodospirillaceae*, *Promicromonosporaceae* were only present in mosquitoes from Ambohidratrimo.

### Molecular characterization of the *Pantoea* isolates

**Figure 2:**
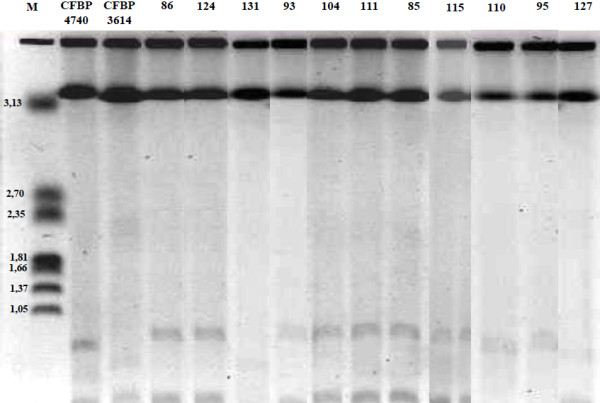
**PFGE of undigested genomic DNA of *Pantoea *mosquito isolates and their reference strains.** Chromosomal DNA from *Hansenula wingei* was used as a reference (BioRad). Characteristics of the samples are indicated in Table 3.

**Figure 3:**
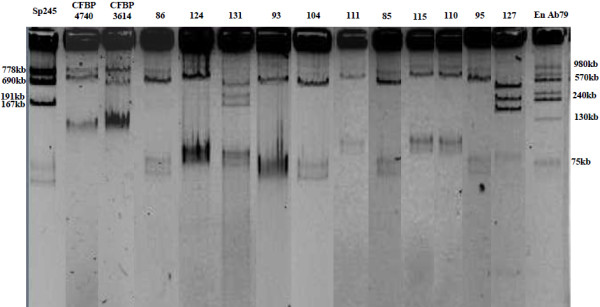
**Electrophoretic profiles of high-molecular-weight plasmids from *Pantoea *mosquito isolates obtained using a modified Eckhardt procedure.** Plasmids from *Azospirillum brazilense* strains En-Ab79 and Sp245 were used as references [38,39]. Characteristics of the samples are indicated in Table 3.

As *Pantoea* was the most prevalent genus isolated from mosquitoes from three of the four sites, it was further characterized by analysing its genomic structure. Nearly complete *rrs* gene sequences were obtained from 11 isolates that were compared to reference strains (Table 3). PFGE showed that *Pantoea* contains a high-molecular-weight replicon (>3.13 Mb), which when considered alongside sequenced reference genomes (http://www.ncbi.nlm.nih.gov) probably corresponds to the bacterial chromosome (Figure 2). Two other replicons each less than 1 Mb were also seen in the PFGE pattern which makes it possible to classify isolates into two groups. One group comprises mosquito isolates no. 127 and no. 131 with the reference strain *Pantoea stewartii* (CFBP 3614), another group included mosquito isolates no. 95 and no. 110 with the reference strain *Pantoea agglomerans* (CFBP 4740) while all other mosquito isolates have patterns closely related to each other but distinct from the reference strains. When the Eckhardt procedure for plasmid analysis was used, high-molecular-weight plasmids (from 75 kb up to 980 kb) from *Pantoea* mosquito isolates were detected. The number (from 2 to 6) and size of plasmids were different from those observed in reference strains (Figure 3). If classified according to plasmid content, mosquito isolates no. 127 and no. 131 showed unique patterns that were similar to each other, while the other mosquito isolates clustered into two distinct groups. The first group included 6 isolates (nos. 85, 86, 93, 95, 104 and 124) and the second group contained 3 isolates (nos. 110, 111 and 115) (Figure 3). Using another method to detect lower-molecular-weight plasmids (less than 28 kb), two supplementary plasmids were detected in mosquito isolates no. 127 and no. 131 only, around 8 and 15 kb (data not shown).

**Table 3:** **Phylogenetic affiliation of *Pantoea *isolates and their 16S rDNA sequences**

	**Name**	**Origin**	**Phylogenetic affiliation**	**Accession numbers**	**Similarity score**^**a **^**(%)**
**Reference strains**	Ref-1	CFBP 474	*Pantoea agglomerans*	U80202	100%
	Ref-2	CFBP 3614	*Pantoea stewartii subsp. indologenes*	FJ611853	100%
**Isolates from *Ae. albopictus***	86	Male, Ankazobe	*Pantoea* sp.	JQ958829	99%
	93	Male, Ankazobe	*Pantoea* sp.	KC217537	96%
	115	Female, Toamasina	*Pantoea* sp.	JQ958827	98%
	124	Female, Toamasina	*Pantoea* sp.	KC217539	99%
	111	Male, Toamasina	*Pantoea* sp.	JQ958826	99%
	127	Male, Toamasina	*Pantoea* sp.	KC217540	99%
	104	Male, Toamasina	*Pantoea* sp.	KC217538	96%
	85	Male, Ankazobe	*Pantoea* sp.	JQ958828	96%
	110	Male, Toamasina	*Pantoea* sp.	JQ958825	97%
	95	Female, Ankazobe	*Pantoea* sp.	JQ958830	97%
	131	Female, Toamasina	*Pantoea* sp.	KC217541	99%

^a^ 16S rRNA gene sequence similarity below 97% may suggest that the isolate represents a new species.

**Figure 4:**
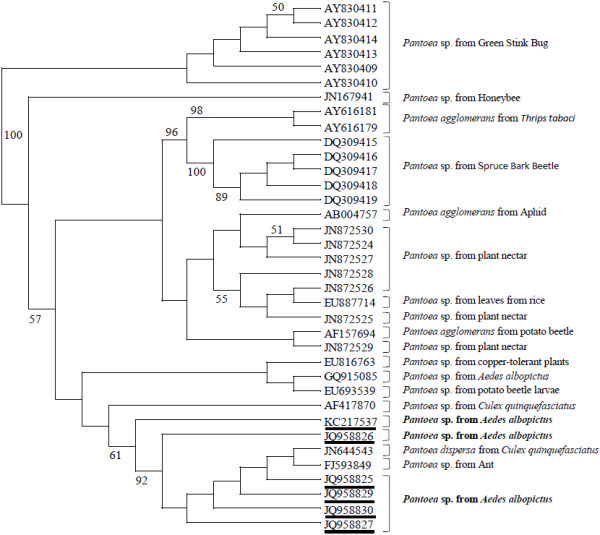
**Phylogenetic analysis based on partial 16S rRNA gene sequences of *Pantoea *obtained from this study and some of those available in GenBank.** Identification and GenBank accession numbers are indicated for each sample. The phylogenetic tree was constructed using the Hasegawa, Kishino and Yano maximum likelihood method, with bootstrap analysis with 1000 replicates. Numbers on branches indicate support for each clade ≥ 50%. Only one representative sequence of *Ae. albopictus Pantoea* isolates is listed in the tree corresponding to the classification of the 45 *Pantoea* isolates according to their ARDRA profile and sequence composition.

One representative sequence of *Pantoea* isolates according to their ARDRA profile and sequence composition was chosen and the sequences were compared to construct a phylogenetic tree. Sequences of *Pantoea* isolated from other insect species (stink bug, honeybee, Onion thrip*,* beetle and the mosquito *Culex quinquefasciatus*) and the environment (plants and plant nectar) (Figure 4) were also included. The topology of the tree showed that *Pantoea* isolated from *Ae. albopictus* clustered with the sequence from *Pantoea dispersa* from *C. quinquefasciatus* and the sequence from *Pantoea* sp. from ant.

## Discussion

We found a total of 27 genera of culturable bacteria associated with the mosquito *Ae. albopictus* caught in different regions of Madagascar. This relatively high number might be partly attributable to the variety of culture media used and provides evidence of the diversity of culturable bacteria present in wild *Ae. albopictus*. The 16S rDNA sequences from the isolates indicated that they belonged to 19 families from three major phyla, Actinobacteria*,* Firmicutes and Proteobacteria. Of the 27 bacterial genera identified here, 12 had been previously found in other mosquito species, so this suggests that these bacteria might be in a close relationship with mosquitoes or enable mosquitoes to be better adapted to the environment [8-10,12,41,42].

Many isolates identified are known to be commensal bacteria for plant and soil environments. In mosquitoes, the origin of commensal bacteria has not yet been fully resolved. Usually bacteria can be acquired in two ways, either by vertical inheritance through generations or through continual acquisition from the environment. Moreover, the mosquito gender is also an important factor that affects bacterial microbiota composition, as already demonstrated [12]. This difference is mainly due to the fact that male and female mosquitoes exhibit different ecological behaviors in terms of nutritional capabilities. Both genders feed on nectar and plant saps, but females are also hematophagous. Consequently, diet regime (sugar and/or blood meals) can significantly affect the bacterial structure. However, information on the sugar feeding of *Ae. albopictus* in the field is scarce [43]. Recently, a first survey of bacteria in floral nectar from a natural plant community showed that *Pantoea* was one of the most common bacterial genus recovered [44]. Interestingly, we showed that this bacterium was also the most prevalent in *Ae. albopictus* mosquitoes, suggesting a potential route of its acquisition through the environment.

A total of eight 16S rDNA sequences identified were similar to those of bacteria encountered in human clinical specimens, including the species *Microbacterium*, *Klebsiella oxytoca* and *Haematobacter massiliensis*[45,46]. As mosquitoes are mostly known to transmit arboviruses and parasites, it is possible that they also transmit, even on a small scale, opportunistic bacterial pathogens to human and animals.

In our previous study of *Ae. albopictus* populations from Madagascar, we identified the phyla Proteobacteria and Firmicutes, with *Bacillus *as a predominant isolated genus [12]. Here the majority of isolates belonged to the *Enterobacteriaceae* family and *Pantoea* was the most common genus probably due to the difference in the sampling region as well as the cultural media used. The relatively high prevalence of *Pantoea* isolates found in the present study emphasizes the need to also consider this bacterium as an intimate partner of the mosquito vector and to better explore its abundance and persistence among field populations, as previously explored in the context of the prevalence study performed on *Acinetobacter* and *Asaia* in the same areas. The genus *Pantoea* is polyphyletic and comprises seven species [47]. Following the results of phylogenetic analyses, sequences of *Pantoea* isolates from *Ae. albopictus* tended to cluster together and with those originated from the *C. quinquefasciatus* species as well as one isolate from ant. A larger number of sequences is thus needed to make conclusions on the presence of well-conserved sequence of *Pantoea* isolates in mosquitoes. For this purpose, it would be necessary to pursue the global effort to obtain new *Pantoea* isolates from insects and environment. Members of *Pantoea* are commonly isolated from the environment, mostly from water and soil, and some isolates have been recovered from human clinical samples or as causative agents of plant diseases. *Pantoea agglomerans* can establish a symbiotic relationship in western flower thrips (*Frankliniella occidentalis*) that persists for over 50 generations or about 2 years [48]. *Pantoea agglomerans* was also the most frequently isolated bacterium from the midgut of *Anopheles funestus* and *An. gambiae* species caught in Kenya and Mali [49], and it has been shown to easily adapt to its hosts [50]. This bacterium was also recently detected in *Ae. albopictus* from North America [51]. Recently, Bisi and Lampe [22] hypothesized that *P. agglomerans* could be engineered to express and secrete anti-plasmodium effector proteins in *Anopheles* mosquitoes. As *Pantoea* was the most prevalent bacterium isolated in our study, it could also be a candidate for paratransgenesis in *Ae. albopictus*. One strategy in paratransgenesis is to insert the gene of interest into plasmids hosted by the chosen bacterium. We found *Pantoea* isolates from *Ae. albopictus* harboured at least two plasmids per genome, so it would be of interest to further determine if plasmids can be laterally transferred between strains and which genes they carry.

## Conclusions

This study highlights the diverse culturable bacteria in field populations of *Ae. albopictus*. Some of them were detected for the first time in this vector and their functions are not known at all. Further studies are needed to investigate the physiological characteristics of the bacterial isolates and their possible interactions with mosquito biology and vector competence. This information could be of great importance in developing new alternative control strategies based on the use of symbiotically modified mosquitoes.

## Competing interests

The authors declare that they have no competing interests.

## Authors’ contributions

CVM conceived the design of the study, participated in all the tasks and performed sequence analysis. FHT carried out the molecular identification of bacteria (ARDRA, PFGE, plasmid profiles). FNR participated in the sampling of mosquitoes and the isolation of bacteria. PR participated in the design of the study. PM conceived of the study and drafted the manuscript. All authors read and approved the final manuscript.
